# Media Multitasking in Mixed Reality Learning Situations: What Determines Its Effectiveness?

**DOI:** 10.11621/pir.2023.0406

**Published:** 2023-12-01

**Authors:** Galina U. Soldatova, Anastasia G. Koshevaya

**Affiliations:** a Lomonosov Moscow State University, Russia

**Keywords:** media multitasking, effectiveness, adolescents, augmented reality, mixed reality, education, eye tracker, metacognition

## Abstract

**Background:**

Media multitasking (MMT) is common among adolescents, especially with the introduction of digital educational tools in mixed reality environments. However, there has been limited research on MMT in educational settings with electronic learning tools including augmented reality (AR).

**Objective:**

To study MMT in conjunction with metacognition, technology attitudes, and effectiveness of learning activities for 13–14 year olds in a mixed reality learning situation.

**Design:**

The experiment involved organizing learning activities in MMT format using digital tools, including AR. The MMT experimental group was given the option of searching the internet for information about a problem; the control group was given only a video, the e-textbook and AR application. Eye tracking measured task switching, while MMT efficiency was assessed by the number of completed tasks and test results. Metacognition was measured using the Metacognitive Awareness Inventory (MAI), and attitudes toward digital devices were examined using the Technology Attitudes Questionnaire.

**Results:**

Most of the adolescents (80%) preferred MMT, and no significant differences in test performance were found between the groups. Multitasking correlated with better cognitive control and metacognition scores and negatively correlated with technophobia. Learning activity effectiveness in mixed reality was assessed by the number and time of fixations on tasks in conjunction with metacognition and cognitive control. Interactive digital tools in education improve learning efficiency.

**Conclusion:**

Adolescents’ preference for multitasking does not reduce learning productivity, but it does not guarantee success either. This suggests an internalization process of using digital technologies among adolescents. As a result, MMT may be gradually mastered as a new tool that is necessary for adaptation and success in an increasingly complex technological reality. Cognitive control and metacognitive planning significantly contribute to MMT efficiency, highlighting the importance of a conscious MMT strategy for effective learning.

## Introduction

Modern life is unthinkable without active use of digital technologies. Russian teenagers are often ahead of adults in their level of activity in the digital environment. In 2019, the average user activity of Russian teenagers was 4–5 hours on weekdays and 6–8 hours on weekends. Compared to 18 other European countries, this is one of the highest levels of weekday user activity (Smahel et al., 2020; Soldatova & Rasskazova, 2023).

High level of internet use defines a new trend in research: online and offline are considered not separately, but as a mixed reality — a single cyberphysical space in which they closely intertwine and interact, organizing people’s daily lives in a new way (Colledani et al., 2023; Floridi, 2015; Skarbez et al., 2021). This unique situation of modern child development became the basis for the analysis of digital childhood in the socio-cognitive concept of digital socialization (Soldatova & Voyskunsky, 2021), which acts as a theoretical framework for our study. This concept is based on the cultural-historical paradigm (Vygotsky, 1960) and the cultural-activity approach developed in Russian psychology (L.S. Vygotsky, A.N. Leontiev, A.G. Asmolov, M. Cole, etc.), the theory of ecosystems by U. Bronfenbrenner (Bronfenbrenner, 1979, 2004), and the theory of expanded consciousness by E. Clark and D. Chalmers (Clark & Chalmers, 1998). The concept postulates the formation in a child of a new ecosystem, including the technosystem — a set of new cultural tools (all digital devices, digital platforms, applications, algorithms, as well as ways to use them). The technosystem, integrating with children’s cognitive, personal, and social systems, mediates their development in mixed reality, transforms the psychological mechanisms of interiorization, exteriorization, and re-exteriorization, and determines the formation of a technologically expanded personality with a new digital sociality. The extended personality masters new activity formats in mixed reality, and one of the most prominent ones is media multitasking (MMT).

According to the cultural-activity approach, there is always an activity between child learning and mental development. One way of using and interacting with digital devices is through MMT. For both children and adults, this format is seen as a new digital sociality that has emerged in response to the increased demands of the environment. Empirical data show that MMT is a common activity format among adolescents (May & Elder, 2018; Soldatova et al., 2020a), which is also penetrating education.

Considering MMT as a type of multitasking involves referring to cognitive psychology, where multitasking has been viewed as simultaneous performance of two or more tasks (Cherry, 1953; Gray & Wedderburn, 1960; Kahneman, 1973; Pashler, 1994). MMT is defined as an activity format that involves performing multiple tasks simultaneously using digital devices. Among the approaches to the study of MMT are the successive and simultaneous approaches (Soldatova et al., 2020b). MMT is also studied as digital distraction (Aagaard, 2019).

### In-class MMT effectiveness

Adolescents actively use digital devices during classes, which has generated much discussion among educators and psychologists (Murphy & Castel, 2023). Some researchers indicate that digital device use in school can negatively impact student achievement (Gray & Schofield, 2021; Wammes et al., 2019) as an indicator of learning effectiveness (May & Elder, 2018; Peifer & Zipp, 2019). Effectiveness, which is also influenced by psychological and situational factors, is one of the key criteria for evaluating MMT. On the one hand, MMT creates an “illusion of productivity”, which can increase motivation and positively impact outcomes. On the other hand, research shows that MMT effectiveness remains illusory (Soldatova et al., 2020b). Despite this, the ability to work with a large number of tasks simultaneously is considered an important supra-professional competence (Zeer et al., 2019). However, the modern educational system usually does not encourage students to multitask, and the pedagogical community doubts the appropriateness of developing this skill (Sidorova, 2021). Distinguishing between negative and positive MMT, researchers associate the negative one with digital distraction of students on their devices, for example, when using messengers during class (Aharony & Zion, 2019; Shane-Simpson & Bakker, 2022), and the positive one with the possibility of searching for additional information (Wu & Xie, 2018). Some studies indicate that there is no direct or indirect relationship between MMT and academic achievement in the long term (van der Schuur et al., 2019).

### MMT and metacognition

Metacognition is the awareness of the characteristics of one’s own cognition and the ability to regulate it, which determines the monitoring of cognitive processes, planning, and development of cognitive strategies, and affects productivity in general (Schraw, 1998). The regulatory component of metacognition is important in learning, as a result of which resources are allocated and concentration on meaningful tasks is achieved. The regulatory component of metacognition may be related to MMT (Soldatova et al., 2020b). In a study by Terry and colleagues (2016), it was found that students with higher metacognition preferred to multitask less, which may identify the self-regulatory nature of multitasking. MMT performance and the ability to manage it are related to metacognition (Murphy & Castel, 2023).

### MMT and cognitive control

Cognitive (executive) control is defined as the cognitive processes that underlie voluntary behavior. Cognitive control includes inhibition (impulse and inhibitory control of automatic responses, self-regulation, and delayed gratification); shifting (task switching, mental attitude change, and cognitive flexibility); and updating (working memory operations) (Aron, 2008; Dreher & Berman, 2002). Research findings on the relationship between MMT and cognitive control are multidirectional. On the one hand, Alzahabi & Becker (2013) found a positive association of MMT with high levels of cognitive control. On the other hand, psychologists at Stanford University (Ophir et al., 2009) showed that heavy multitaskers find it more difficult to suppress irrelevant information. And Baumgartner et al. (2014) found no relationship between MMT and executive function, but multitaskers reported problems with self-regulation in everyday life when self-assessing.

### MMT and digital technology attitudes

Involvement in MMT determines the intensity of interaction with the technosystem and is inextricably linked to attitudes towards digital technologies. People with positive attitudes tend to be more technologically equipped in their environment and, consequently, live to a greater extent in a mixed reality, which implies constant switching between different worlds. A positive relationship between MMT and technophilia as an indicator of openness and enthusiasm for using digital devices has been found in a number of studies (Cotten et al. 2014; Ettinger & Cohen 2020). Researchers have also linked students’ MMT to fear of missing out (FOMO), when a person is afraid of missing something important and constantly checks their phone (Shane-Simpson, Bakker, 2022; Terry et al., 2016).

### Digital tools in education (augmented reality — AR)

Researchers in the field of pedagogy and psychology pay attention to the use of digital technologies in the educational environment in general (Tserkovnikova & Tretiakova, 2021; Uvarov, 2018). In particular, the possibilities and prospects of mixed reality learning, using augmented or virtual reality technologies, are considered. AR technologies are becoming an educational tool, if not in schools, then in museums, encyclopedias, and individual programs. It is important to consider the opportunities that AR offers for education. Research findings show that learning in mixed reality arouses students’ interest, increases their motivation and engagement in the learning process. Moreover, AR-based learning has been found to have a positive effect on learning outcomes (Maas & Hughes, 2020).

### Methods of studying MMT

The most common instruments for studying MMT are the Media Multitasking Index (Ophir et al., 2009) and the Short Media Multitasking Measure for adolescents (Baumgartner et al., 2017). These questionnaires are based on participants’ self-assessment of their media consumption patterns. However, research has shown that most users incorrectly estimate their screen time and MMT (Júdice et al., 2023; Soldatova et al., 2022). Looking for a more objective MMT assessment, we developed and tested a quasi-experimental study design that replicates the situation of everyday MMT in children and adolescents. The quasi-experiment included simultaneous performance of several tasks on a computer and a smartphone (Soldatova et al., 2020a).

### Research problem

Digital technologies, particularly AR, are becoming more and more accessible and are penetrating the education system. This contributes to the transition of life into a mixed reality, which requires individuals to interact with the environment in new ways. One way is the MMT format, which is especially prevalent among adolescents and has become a key characteristic of today’s successful person (Zeer et al., 2019). Despite the demands of the environment, school as the main socialization institution does not provide the necessary tools for the development of this soft skill, so teenagers master this format chaotically, to the detriment of their effectiveness.

The purpose of the present study was to investigate MMT features in conjunction with metacognition, technology attitudes, and learning activities effectiveness in 13–14-year-old students in a learning situation using digital tools, including AR.

*Hypotheses:*

**H1:** number and time of fixations as indicators of the task execution strategy predict the effectiveness of schoolchildren’s learning activities in the MMT format.

**H2:** higher metacognition is associated with shorter fixation time on tasks and higher number of fixations.

**H3:** higher level of cognitive control is associated with less time and greater number of fixations on tasks.

**H4:** technophobia as an indicator of negative attitudes towards digital technology negatively correlates with the number of fixations and positively with fixation time.

**H5:** number and time of fixations in conjunction with high levels of cognitive control and metacognition predict the effectiveness of learning activities in the MMT format.

**H6:** the additional use of online search and an AR app in a digital learning situation enhances the effectiveness of learning activities in the MMT format.

## Methods

**Figure 1.**
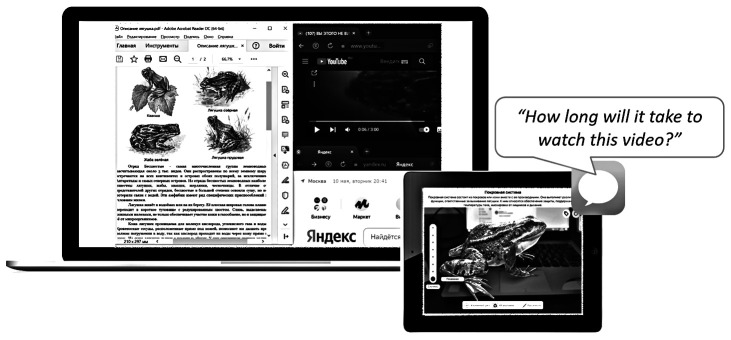
Tasks on the computer and tablet

1. A specially designed experiment used a computer and tablet to assess behavioral features of learning task performance in MMT conditions. There were six biology tasks that took 10 minutes to complete: to find out why frogs are called cold-blooded; to study frog life-cycle stages; what helps frogs alive both in water and on land; to learn information about frog anabiosis; to study the frog skeleton and digestive system. To simulate distraction conditions in a learning situation, adolescents received an SMS during the tasks. Participants self-tracked the time at the bottom of the screen. The number and content of the tasks were selected so that they required more than the allotted time to complete. Participants were divided into two groups: experimental and control. For the experimental group, the instruction mentioned the possibility of using online search, while the control group did not. Participants received a computer with two or three windows (in the control group, windows with a video about frog anabiosis and text about amphibians from the e-textbook; in the experimental group, the same two windows and a third window with a browser for searching) and a tablet (studying the anatomy and life cycle of frogs in augmented reality) (*Figure 1*). An eye tracker was used to record the number and time of fixations on each computer window or tablet. The experiment included an observation method, which documented the adolescent’s behavior in the process of performing tasks: refusal to perform tasks, overwork, etc.
2. Two indicators were defined to evaluate MMT effectiveness: 1) the score on a control test to check the knowledge obtained during the experiment (0–9 points); 2) the number of completed tasks given in the instruction (0–5 points): watching a video, reading text, answering SMS, studying frog anatomy and life cycle in AR.
3. A structured interview with questions about attitudes toward the e-learning tools (e.g., “Did you enjoy working with the AR app?”, “Which would you prefer: textbook or app?”) and multitasking in education (“Would you like various lessons to be taught in a format where information could be obtained from different sources (app, textbook, video, audio lecture, notes)?”).
4. A modified computerized test “Dots: Hearts & Flowers” (Korneev et al., 2018) was used to assess executive function performance.
5. Metacognition was measured using a modified Metacognitive Awareness Questionnaire (MAI) (Karpov & Skityaeva, 2005).
6. Attitudes toward digital devices were assessed using the Technology Attitudes Questionnaire (Soldatova et al., 2021).

### Participants

The study involved 64 eighth-grade students (37 girls and 27 boys) aged 13–14 from three Moscow schools.

### Materials

- Laptop computer with stimuli. Stimuli were presented on a 15.6” LED monitor with a 1920x1080 FHD resolution LCD monitor located 75 cm from the observer’s head;
- A tablet with the “Froggipedia” AR program developed by Indiavidual Learning Limited;
- Eye movements during the experiment were recorded in binocular mode using a Pupil Labs Core eye tracker with a frequency of 200 Hz and < .02° resolution.

### Procedure

The study was conducted in a school or laboratory setting. First, the participant filled out a questionnaire that included sociodemographic questions, the Technology Attitude Questionnaire, and the MAI. An eye tracker was then worn and calibrated to record eye movements during the experiment. Participants received verbal and paper instructions that required them to learn the biology topics within a limited time. The experimenter emphasized that there were many tasks and only 10 minutes, and then showed how to work with the AR app. Thus, using different devices, participants had to complete several learning tasks in 10 minutes. After 5 minutes, a message with an additional task was sent to the tablet. After 10 minutes, the experimenter stopped the task, gave a 3-minute test, and interviewed the adolescents. Participants then performed the “Dots” test.

Statistical analysis was conducted using IBM SPSS Statistics v. 22 and Jamovi. Methods of descriptive statistics, F-test, correlation, regression and mediator analyses were used.

## Results

### MMT strategies and effectiveness

**Figure 2.**
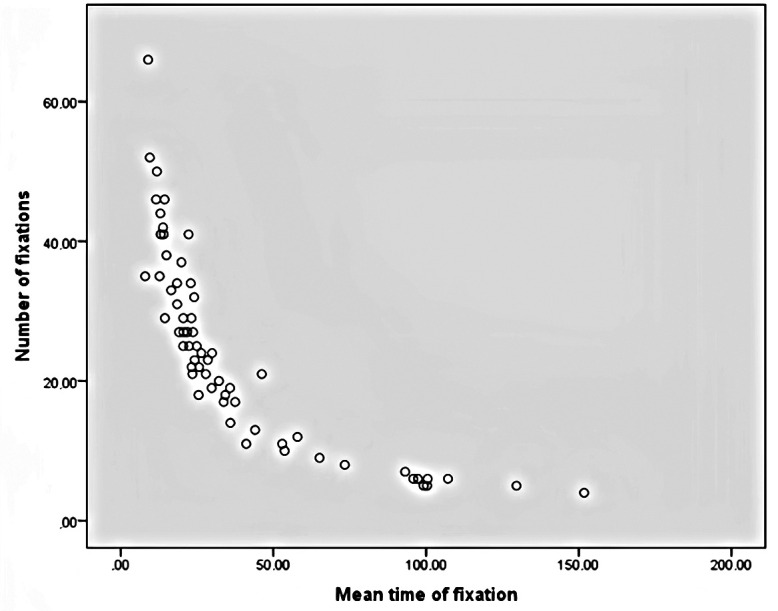
Scatter plot of the relationship between mean fixation time (sec) and number of fixations.

The variables number and time of fixations have a negative non-linear relationship: the more fixations, the shorter their average time (*rho* = –.959; *p* < .001) (*Figure 2*). The bulk of observations are concentrated in the region of 23.52 ± 7.81 fixations, with an average fixation time of 28.61 ± 12.54 seconds (44 participants). A cluster of observations stands out, according to which participants fixated longer on tasks (*M* = 104.82 ± 21.48 sec), making a minimal number of switches (*M* = 5.8 ± 1.14) (nine participants). The other cluster, in the upper left corner of the diagram, also includes nine people, who were characterized by a shorter fixation time (*M* = 13.26 ± 3.65 sec) with a greater number (*M* = 46.9 ± 7.74).

Group separation made it possible to assess the effectiveness of learning activities at different MMT levels; there were no significant differences.

In addition to eye tracker indicators, MMT was also considered as a digital distraction. During the experiment, only 21% of participants looked at the message sent to them and only 11% responded to the text message by performing an additional task. Texting distraction did not affect control test results (*Table 2*).

A correlation analysis of the relationship of the number and time of fixations with the MAI, Technology Attitude Questionnaire, and “Dots” test results was carried out (*Table 1*).

**Table 1:** Correlations of mean fixation time and number of fixations with MAI, Technology Attitude Questionnaire, Dots: Hearts & Flowers (HF) test^1^

	Number of switches	Mean time of fixations (sec)
	*R*	
Information Management Strategies	.269^*^	–.392^**^
Regulation of Cognition	.221	–.278^*^
Technophobia	–.151	.278^*^
Correct answer reaction time in tries F (second trial), sec	–.224	.386^**^
Correct answer reaction time in tries HF (third trial), sec	–.389^**^	.370^**^
Number of correct answers in tries HF	–.283^*^	.200
Average reaction time, s	–.242	.351^*^
Average reaction time in tries F, s	–.221	.381^**^
Average reaction time in tries HF, s	–.324^*^	.340^*^
Correct answer reaction time, s	–.271	.372^**^
Number of errors, Flowers (second trial)	–.330^*^	.407^**^

* p < .05; ** p < .01

### Cognitive control and performance strategy in learning MMT situation

The number of fixations negatively correlated with reaction time in correct response (*r* = –.389; *p* = .005) and total reaction time (*r* = –.324; *p* = .021) in the “Dots” third trial: those who switch more had a higher level of cognitive control. There was also negative correlation between the number of fixations and the number of errors in the “Dots” second trial (*r* = –.330; *p* = .018): those who switched more had fewer errors, indicating a better ability to switch from one instruction to another. Longer fixation duration was associated with increased correct answer reaction time in the whole Dots test (*r* = .351; *p* = .012), as well as in the second (*r* = .386; *p* = .005) and third (*r* = .370; *p* = .008) trials. The same regularity was found when considering total reaction time in the whole test (*r* = .351; *p* = .012), as well as in the second (*r* = .381; *p* = .006) and third (*r* = .34; *p* = .015) trials, which may indicate a greater switching difficulty when performing two concurrent programs. In addition, longer fixation duration was positively associated with the number of errors in the second trial (*r* = .407; *p* = .003), suggesting worse cognitive control.

### Metacognition and performance strategy in learning MMT situation

The number of fixations was positively correlated with the Information Management Strategies scale of the MAI (*r* = .269; *p* = .035) and at a sub-significant level with the Regulation of Cognition scale (*r* = .221; *p* = .084). The mean fixation time on the task was negatively correlated with the Information Management Strategies scale (*r* = –.392; *p* = .002).

A correlation analysis between metacognition and cognitive control was also conducted. A higher score on the Information Management scale was associated with faster reaction time overall on the “Dots” test (*r* = –.366; *p* = .008) and in the first (*r* = –.392; *p* = .004) and second (*r* = –.36; *p* = .01) trials. Also at the sub-significant level, faster reaction time for correct answers in the second trial was associated with a higher total metacognition score (*r* = –.249; *p* = .078).

### Technology attitudes and performance strategy in learning MMT situation

Mean fixation time positively correlated with the Technophobia scale (Technology Attitude Questionnaire) (*r* = .278; *p* = .029).

### Educational tools and learning efficiency in MMT

The instruction regarding search use in the experimental group was rather flexible — it did not compel the participants to search for information on the internet, so not all of them used this opportunity. Three strategies of search use were identified: 1) supplementing the presented materials by searching for information on the internet (15 people); 2) substituting familiarization with the presented materials with searching for information on the internet (two people); 3) did not use the search (14 people). Variance analysis revealed differences in performance: those who substituted familiarity with the submitted materials with internet searches were less effective than those who used the other two search utilization strategies (*F* = 3.158; *p* = .048) (*Table 2*).

**Table 2:** Influence of situational factors on the effectiveness of activities in MMT

Factor	Group	*N*	*F*	M	SD
SMS Distraction	Distracted	13	.013	5.451	1.76
	Not distracted	49		5.385	1.8
Online search usage strategy	Enhancement	15	3.158*	4.8	1.76
	Substitution	2		2.5	0.7
	Not used	14		5.533	1.6
AR app usage	Used	52	3.425	5.615	1.75
	Not used	10		4.667	1.61

* p < .05

The analysis of test performance depending on the use of AR showed that the majority of the adolescents chose to work with the application (78.7%) and at the trend level obtained higher test scores (*F* = 3.425; *p* = .069). These empirical facts allow us to confirm hypothesis 6.

### MMT effectiveness predictors

A hierarchical regression analysis was applied ( *Table 3*). First, the hypothesis was tested whether number and time of fixations were predictors of effectiveness in the MMT format. The constructed model showed no significant contribution of these factors (*R*^2^ = .0278; *p* = .435) and their interaction (*R*^2^ = .0283; *p* = .641) (Model 1, 2).

**Table 3:** Regression models of predictors of learning effectiveness in the MMT format

Model	R^2^	P
1	.0278	.435
2	.0283	.641
3	.0692	.179
4	.213	.048
5	.214	.086

**Table 4:** Model 4: predictors of learning effectiveness in the MMT

Predictor	*P*	*β*
Intercept	.040	
Number of fixations	.521	–.142
Average fixation time	.479	.154
Dots: Total number of correct answers	.011	.686
Dots: Number of correct answers (third trial)	.023	–.613
Metacognitive planning	.068	–.257

Next, we tested the hypothesis about the significance of MMT strategy (number and time of fixations on tasks) in combination with cognitive control and metacognition. As an indicator of cognitive control, we took the productivity of the Dots test (total score and third trial). The MAI total score was taken as an indicator of meta-cognition. The regression analysis showed that the model did not explain the contribution of these factors to MMT effectiveness (*R*^2^= .069; *p* = .179) (Model 3). Further exclusion of the total metacognition score from the model and stepwise inclusion of the MAI questionnaire scales in the model showed an improvement in the model with the inclusion of the Planning scale (Model 4) (*R*^2^= .213; *p* = .048). Excluding any of the indicators from the model worsened it. Including the interaction factor of fixations number and time in the model improved the proportion of explained variance by 1%, but made the model significant at the trend level (*R*^2^= .214; *p* = .086) (Model 5). Thus, the best model of predictors of learning effectiveness in MMT format is Model 4: metacognitive planning (β = –.257; *p* = . 068), cognitive control (Dots: total number of correct answers (β = .686; *p* = .011), number of correct answers in third trial (HF) (β = –.613; *p* = .023) and MMT format performance strategy (number of fixations (β = –.142; *p* = .521) and fixation time (β = .154; *p* = .479)).

The most significant factor in MMT effectiveness is cognitive control, while metacognitive planning is significant at the trend level. The number of fixations and their average time do not have unique contributions to effectiveness, but they are related to it in a certain way together with the variables listed above.

Mediator analysis showed no significant results (*p* > .05).

## Discussion

### Performance strategies in a learning MMT situation in mixed reality

Two small, equal groups were identified, which can be categorized as single-taskers and “heavy” multi-taskers. The third and largest group (71%) comprised adolescents who preferred such a strategy of multitasking in a learning situation, where the average number of fixations is 23.52 with an average fixation time of 28.61 seconds. We tentatively name this group “average” multitaskers (Murphy et al., 2017), as its indicators stand out when comparing it with the two extremes, “heavy” and “single-taskers”, whose average number of fixations was 46.9 and 5.8, respectively, and average fixation times were 13.26 and 104.82 seconds. The results support findings on the prevalence of multitasking strategy among the younger generation, using it to adapt to mixed reality (Colledani et al., 2023). Adolescents more often choose “average” multitasking strategy, and the percentage of “heavy” multitaskers appears to be small, which is consistent with other studies (Soldatova et al., 2020a). In the current study, no differences in performance were found between single-taskers, “average”, and “heavy” multitaskers. Similar results have been reported in other studies where multitasking preference did not always affect performance (Kirschner & De Bruyckere, 2017; van der Schuur et al., 2019). This indicates that each child intuitively chooses the appropriate degree of multitasking for him/herself depending on their abilities and resources.

### Cognitive control and performance strategies in a learning MMT situation

Research on the relationship between MMT and cognitive control is mixed: there are results indicating rather a negative correlation between the two (Ophir et al., 2009); another study found no such correlation (Baumgartner et al., 2014), while another group of papers reported a positive one (Alzahabi & Becker, 2013; Matthews et al., 2022). Our results also demonstrated that MMT preference is associated with cognitive benefits (more developed cognitive control). In such a case, a small number of switches and long fixation time could be considered as “getting stuck” on a task. This may indicate poorer functioning of cognitive control, which is particularly involved when working in such a resource-intensive activity format as MMT.

### Metacognition and performance strategies in a learning MMT situation

Most researchers agree that metacognition negatively correlates with MMT (May & Elder, 2018; Peng & Tullis, 2021; Terry et al., 2016). However, there are studies that have not found a negative effect of multitasking on metacognition (Konishi et al., 2020). In our study, a more multitasking learning strategy was associated with higher metacognition scores in the context of information management. Adolescents who chose a more multitasking strategy showed better skills and strategy sequences used to process information more efficiently (e.g., organizing, elaborating, summarizing, selective focusing). Parry and le Roux (2019) also discussed the development of metacognition as a way to manage multitasking, but in this context, “multitasking management” is understood as reducing the degree of multitasking.

### Technology attitudes and performance strategy in the MMT situation

Adolescents who preferred a more single-tasking performance strategy were characterized by more negative attitudes towards digital technologies. This is consistent with research findings in which greater student multitasking was associated with positive attitudes toward digital technology (Cotten et al., 2014; Ettinger & Cohen, 2020; Shane-Simpson & Bakker, 2022; Terry et al., 2016). Such results may indicate greater integration of multitaskers into the technosystem through positive attitudes toward digital devices. Thus, MMT acts as a way to enhance the process of integration with the technosystem (Soldatova & Voyskunsky, 2021), where the real (“physical”) and virtual (“digital”) worlds intertwine, creating a “phygital” world in which the ability to direct one’s attention to several different sources of information is essential (Colledani et al., 2023). This is also supported by research on the relationship between MMT, performance, and cognitive control at different ages: the highest MMT performance was observed among the younger generation (7–27 years old), which may be related to their MMT training due to the abundance of digital technologies accompanying their development (Matthews et al., 2022).

### Educational tools and learning activity efficiency in MMT

According to our study, the majority of adolescents would like modern educational tools to be included in the learning process. This is consistent with results of a study on VR- and AR-technologies usage in biology lessons. The majority of participants positively evaluated the experience, noting AR’s accessibility and interactivity (Garcia-Bonete et al., 2019). Our study extends the understanding of the use of digital tools in the learning environment and shows that their use as a complement to the educational process, rather than as a substitute for it, not only does not reduce the effectiveness of learning, but in some cases even improves it. This is confirmed by the higher test results of teenagers who used the AR application.

### Activity effectiveness predictors in learning MMT situation

According to the results of our study, single-taskers and multitaskers did not differ in activity efficiency. This can be explained by the experimental instruction, which did not oblige multitasking. Adolescents independently chose their performance strategy. Kononova et al. (2016) demonstrated that being compelled to multitask leads to inefficiency. People can only be effective in MMT when they self-regulate their actions. Regulating one’s action strategy is generally possible in everyday life, but is often limited in the laboratory, which may partially explain the inconsistency of our results with most studies concluding that MMT harms performance (Aharony & Zion, 2019; Gray & Schofield, 2021; Wammes et al., 2019). Those studies, however, do not always address the mediating factors that may predict MMT efficacy. In order to identify such factors, we conducted a regression analysis, which suggested that for effective work in MMT it is not so much the performance strategy (number and time of fixations) that is important, but rather a high level of cognitive control and metacognitive planning. Based on these resources, the adolescent chooses the optimal combination of the number of switches and the fixation time on each task. The most significant predictor of MMT effectiveness was found to be a high level of cognitive control. This coincides with the results of studies in which MMT is associated with better performance of executive functions (Alzahabi & Becker, 2013; Matthews et al., 2022). Along with cognitive control, metacognition plays an important role in MMT performance, which is one of the pathways (mediators) through which successful multitasking occurs (Fazeli et al., 2017). The correlation between metacognition and cognitive control, as well as their co-impact on MMT performance, suggests that the interaction between cognitive control and metacognition may be a mediating factor of MMT performance in a learning situation. It was shown that the integration of metacognition and cognitive control can improve children’s perception and management of their own learning (Marulis et al., 2020). However, our mediator analysis did not reveal such an effect, although this may be due to sample size. Further research could focus on the effects of metacognition on cognitive control and the possibility of developing metacognition to improve MMT performance in adolescents.

## Conclusion

The study showed the prevalence of a multitasking strategy among adolescents in a learning situation saturated with electronic learning tools. MMT to a greater extent was determined not so much by the activity associated with the use of digital devices, as with the need to solve different tasks under conditions of limited time resources. However, given three different styles of adolescents’ organization of their learning activities in mixed reality (from minimal switching and prolonged focus on tasks to chaotic switching and short fixation on tasks), no significant differences between groups were obtained in the efficiency of solving the problems on the final test. It is possible that teenagers’ preference for a multitasking strategy, on the one hand, does not harm the productivity of learning activities, but on the other hand, the choice of such a format does not lead to success. This may indicate a process of internalization of this way of using various digital technologies and tools, which is taking place at this stage among adolescents who prefer MMT. As a result, multitasking may be gradually mastered as a new tool that is necessary for adaptation and success in an increasingly complex technological reality. The success of this process supposes that the adolescent has an interest in digital devices, while technophobia may hinder it.

Cognitive control and metacognitive planning make the greatest contribution to the effectiveness of MMT activities. The results show that the productivity of learning activities can be ensured by the conscious choice of an MMT strategy that best suits each adolescent, depending on his or her cognitive and metacognitive abilities related to the voluntary regulation of activities. Focusing on the development of these two components in adolescents may allow them to cope with the intensive information flow, using digital devices and ways of interacting with them as educational tools to facilitate better learning.

## Limitations

This study’s limitations include the small sample size and the narrow age range of participants (13–14 years old), which hinders generalizing the results and warrants further research across different age groups with larger sample sizes. Additionally, the absence of similar studies using AR technology in the educational MMT environment restricts the ability to compare the data with other research, leading to separate comparisons with MMT studies in education and AR in education. Lastly, the possibility that the instruction might have influenced the teenagers’ choice of activity strategy is worth considering, due to its potential impact on the outcomes.

## Footnotes

1 Only significant correlations are shown; full table is in the appendix.

## Appendix

**Table:** Correlations of mean fixation time and number of fixations with MAI, Technology Attitude Questionnaire, Dots: Hearts & Flowers (HF) test, academic performance and effectiveness

	Number of switchings	Mean time of fixations
	*R*	
Declarative Knowledge	.099	–.110
Procedural Knowledge	.003	–.007
Conditional Knowledge	.150	–.095
Knowledge about Cognition	.102	–.088
Planning	.102	–.198
Information Management Strategies	.269^*^	–.392^**^
Comprehension Monitoring	.145	–.086
Debugging Strategies	.230	–.169
Evaluation	.153	–.233
Regulation of Cognition	.221	–.278^*^
MAI Total	.193	–.227
Technophilia	.066	–.186
Technorationalism	.099	–.195
Technophobia	–.151	.278^*^
Technopessimism	.027	.062
Correct answer reaction time in tries H (first trial), sec	–.086	.216
Correct answer reaction time in tries F (second trial), sec	–.224	.386^**^
Correct answer reaction time in tries HF (third trial), sec	–.389^**^	.370^**^
Number of correct answers in tries H	–.121	.090
Number of correct answers in tries F	.034	–.109
Number of correct answers in tries HF	–.283^*^	.200
Average reaction time, sec	–.242	.351^*^
Average reaction time in tries H, sec	–.080	.209
Average reaction time in tries F, sec	–.221	.381^**^
Average reaction time in tries HF, sec	–.324^*^	.340^*^
Correct answer reaction time, sec	–.271	.372^**^
Number of correct answers	–.153	.080
Number of incorrect answers	.049	.016
Number of incorrect answers in tries H	.021	–.059
Number of errors F	–.330^*^	.407^**^
Number of incorrect answers in tries HF	.229	–.149
Number of missed responses	–.034	.138
Number of missed responses in tries H	–.012	.205
Number of missed responses in tries F	–.105	.176
Number of missed responses in tries HF	.012	–.046
Functional state change	–.189	.205
Mood change	–.028	.086
Academic perfomance	–.037	.027
Effectiveness	.166	–.125

** p < .05; ** p < .01*

## References

[ref1] Aagaard, J. (2019). Multitasking as distraction: A conceptual analysis of media multitasking research. Theory & Psychology, 29(1), 87–99. 10.1177/0959354318815766

[ref2] Aharony, N., & Zion, A. (2019). Effects of WhatsApp’s use on working memory performance among youth. Journal of Educational Computing Research, 57(1), 226–245. 10.1177/0735633117749431

[ref3] Alho, K., Moisala, M., & Salmela-Aro, K. (2022). Effects of media multitasking and video gaming on cognitive functions and their neural bases in adolescents and young adults. European Psychologist, 27(2), 131–140. 10.1027/1016-9040/a000477

[ref4] Alzahabi, R., & Becker, M.W. (2013). The association between media multitasking, task-switching, and dual-task performance. Journal of Experimental Psychology: Human Perception and Performance, 39(5), 1485. 10.1037/a0031208 23398256

[ref5] Aron, A.R. (2008). Progress in executive-function research: From tasks to functions to regions to networks. Current Directions in Psychological Science, 17(2), 124–129. 10.1111/j.1467-8721.2008.00561.x

[ref6] Baumgartner, S.E., Weeda, W.D., van der Heijden, L.L., & Huizinga, M. (2014). The relationship between media multitasking and executive function in early adolescents. The Journal of Early Adolescence, 34(8), 1120–1144. 10.1177/027243161452313

[ref7] Bronfenbrenner, U. (1979). The ecology of human development: Experiments by nature and design. Harvard University Press.

[ref8] Bronfenbrenner, U. (Ed.). (2004). Making human beings human: Bioecological perspectives on human development. Sage Publ.

[ref9] Butt, N., & Warraich, N.F. (2022). Multitasking behavior in the workplace: A systematic review. Journal of Social Research Development, 3(2), 229–247. 10.53664/jsrd/03-02-2022-08-229-247

[ref10] Cherry, E.C. (1953). Some experiments on the recognition of speech, with one and with two ears. The Journal of the Acoustical Society of America, 25(5), 975–979. 10.1121/1.1907229

[ref11] Clark, A., & Chalmers, D. (1998). The extended mind. Analysis, 58(1), 7–19. 10.1093/analys/58.1.7

[ref12] Colledani, D., Anselmi, P., & Robusto, E. (2023). Development of a scale for capturing psychological aspects of physical–digital integration: relationships with psychosocial functioning and facial emotion recognition. AI & Society, 1–13. 10.1007/s00146-023-01646-9 PMC10031718 37358941

[ref13] Cotten, S.R., Shank, D.B., & Anderson, W.A. (2014). Gender, technology use and ownership, and media-based multitasking among middle school students. Computers in Human Behavior, 35, 99–106. 10.1016/j.chb.2014.02.041

[ref14] Dreher, J.C., & Berman, K.F. (2002). Fractionating the neural substrate of cognitive control processes. Proceedings of the National Academy of Sciences of the United States of America, 99(22), 14595–14600. 10.1073/pnas.222193299 12391312 PMC137928

[ref15] Ettinger, K., & Cohen, A. (2020). Patterns of multitasking behaviours of adolescents in digital environments. Education and Information Technologies, 25, 623–645. 10.1007/s10639-019-09982-4

[ref16] Fazeli, P.L., Casaletto, K.B., Woods, S.P., Umlauf, A., Scott, J.C., Moore, D.J., & HNRP Group. (2017). Everyday multitasking abilities in older HIV+ adults: Neurobehavioral correlates and the mediating role of metacognition. Archives of Clinical Neuropsychology, 32(8), 917–928. 10.1093/arclin/acx047 28575231 PMC5860015

[ref17] Floridi, L. (2015). The onlife manifesto: Being human in a hyperconnected era (p. 264). Springer Nature. 10.1007/978-3-319-04093-6

[ref18] Garcia-Bonete, M.J., Jensen, M., & Katona, G. (2019). A practical guide to developing virtual and augmented reality exercises for teaching structural biology. Biochemistry and Molecular Biology Education, 47(1), 16–24. 10.1002/bmb.21188 30475432

[ref19] Gray, J.A., & Wedderburn, A.A.I. (1960). Shorter articles and notes grouping strategies with simultaneous stimuli. Quarterly Journal of Experimental Psychology, 12(3), 180–184. 10.1080/1747021600841672

[ref20] Gray, J., & Schofield, D. (2021). Media multitasking: A cross-cultural study. International Journal of Computer Trends and Technology, 69(3), 64–73. 10.14445/22312803/IJCTT-V69I3P112

[ref21] Júdice, P.B., Sousa-Sá, E. & Palmeira, A.L. (2023) Discrepancies between self-reported and objectively measured smartphone screen time: Before and during lockdown. J of Prevention, 44, 291–307 10.1007/s10935-023-00724-4 PMC9872730 36692818

[ref22] Kahneman, D. (1973). Attention and effort (Vol. 1063, pp. 218–226). Prentice-Hall.

[ref23] Karpov, A.V., & Skityaeva, I.M. (2005). Psikhologiia metakognitivnykh protsessov lichnosti [Psychology of metacognitive processes of personality]. Izdatel’stvo Institut psihologii RAN [Publishing House of the Institute of Psychology of the Russian Academy of Sciences].

[ref24] Kirschner, P.A., & De Bruyckere, P. (2017). The myths of the digital native and the multitasker. Teach. Teach. Educ. 67, 135–142. 10.1016/j.tate.2017.06.001

[ref25] Konishi, M., Compain, C., Berberian, B., Sackur, J., & de Gardelle, V. (2020). Resilience of perceptual metacognition in a dual-task paradigm. Psychonomic Bulletin & Review, 27, 1259–1268. 10.3758/s13423-020-01779-8 32705620

[ref26] Kononova, A., Joo, E., & Yuan, S. (2016). If I choose when to switch: Heavy multitaskers remember online content better than light multitaskers when they have the freedom to multitask. Computers in Human Behavior, 65, 567–575. 10.1016/j.chb.2016.09.011

[ref27] Korneev, A., Akhutina, T., Gusev, A., Kremlev, A., & Matveeva, E. (2018). Computerized neuropsychological assessment in 6–9 years-old children. KnE Life Sciences, 495–506. 10.18502/kls.v4i8.3307

[ref28] Maas, M.J., & Hughes, J.M. (2020). Virtual, augmented and mixed reality in K–12 education: A review of the literature. Technology, Pedagogy and Education, 29(2), 231–249. 10.1080/1475939x.2020.1737210

[ref29] Marulis, L.M., Baker, S.T., & Whitebread, D. (2020). Integrating metacognition and executive function to enhance young children’s perception of and agency in their learning. Early Childhood Research Quarterly, 50, 46–54. 10.1016/j.ecresq.2018.12.017

[ref30] Matthews, N., Mattingley, J.B., & Dux, P.E. (2022). Media-multitasking and cognitive control across the lifespan. Scientific Reports, 12(1), 4349. 10.1038/s41598-022-07777-1 35288584 PMC8919358

[ref31] May, K.E., & Elder, A.D. (2018). Efficient, helpful, or distracting? A literature review of media multitasking in relation to academic performance. International Journal of Educational Technology in Higher Education, 15(1), 1–17. 10.1186/s41239-018-0096-z

[ref32] Murphy, D.H., & Castel, A.D. (2023). Responsible attention: The effect of divided attention on metacognition and responsible remembering. Psychological Research, 87(4), 1085–1100. 10.1007/s00426-022-01711-w 35838835 PMC10191991

[ref33] Murphy, K., McLauchlan, S., & Lee, M. (2017). Is there a link between media-multitasking and the executive functions of filtering and response inhibition? Computers in Human Behavior, 75, 667–677. 10.1016/j.chb.2017.06.001

[ref34] Ophir, E., Nass, C., & Wagner, A. D. (2009). From the cover: Cognitive control in media multitaskers. Proceedings of the National Academy of Sciences of the United States of America, 106(37), 15583. 10.1073/pnas.0903620106 19706386 PMC2747164

[ref35] Parry, D.A., & le Roux, D.B. (2019). Media multitasking and cognitive control: A systematic review of interventions. Computers in Human Behavior, 92, 316–327. 10.1016/j.chb.2018.11.031

[ref36] Pashler, H. (1994). Dual-task interference in simple tasks: data and theory. Psychological Bulletin, 116(2), 220–244. 10.1037/0033-2909.116.2.220 7972591

[ref37] Peifer, C., & Zipp, G. (2019). All at once? The effects of multitasking behavior on flow and subjective performance. European Journal of Work and Organizational Psychology, 28(5), 682–690. 10.1080/1359432x.2019.1647168

[ref38] Peng, Y., & Tullis, J.G. (2021). Dividing attention impairs metacognitive control more than monitoring. Psychonomic Bulletin & Review, 28(6), 2064–2074. 10.3758/s13423-021-01950-9 34131889 PMC8205317

[ref39] Schraw, G. (1998). Promoting general metacognitive awareness. Instructional Science, 26, 113–125. 10.1023/a:1003044231033

[ref40] Shane-Simpson, C., & Bakken, T. (2022). Students’ fear of missing out predicts in-class social media use. Teaching of Psychology, 0(0). 10.1177/00986283211060752

[ref41] Sidorova, T.V. (2021). Mul’tizadachnost’ sovremennogo pedagoga: mif ili real’nost’ [Multitasking of a modern teacher: myth or reality]. Vestnik Buriatskogo gosudarstvennogo universiteta. Obrazovanie. Lichnost’. Obshchestvo. [Bulletin of the Buryat State University. Education. Personality. Society], 3, 10.18101/2307-3330-2021-3-48-52

[ref42] Skarbez, R., Smith, M., & Whitton, M. C. (2021). Revisiting Milgram and Kishino’s reality-virtuality continuum. Frontiers in Virtual Reality, 2, 647997. 10.3389/frvir.2021.647997

[ref43] Smahel, D., Machackova, H., Mascheroni, G., Dedkova, L., Staksrud, E., Ólafsson, K. , ... & Hasebrink, U. (2020). EU Kids Online 2020: Survey results from 19 countries. 10.21953/lse.47fdeqj01ofo

[ref44] Soldatova G.U., & Vojskunskij, A.E. (2021). Sotsial’no-kognitivnaia kontseptsiia tsifrovoi sotsializatsii: novaia ekosistema ili evoliutsiia psikhiki [The socio-cognitive concept of digital socialization: A new ecosystem and the social evolution of the psyche, Psikhologiia. Zhurnal Vysshei shkoly ekonomiki [Psychology. Journal of the Higher School of Economics], 18(3), 431–450. 10.17323/1813-8918-2021-3-431-450

[ref45] Soldatova, G.U., Nestik, T.A., Rasskazova, E.I., Dorokhov, E.A. (2021). Psikhodiagnostika psikhofobii i tekhnofilii: razrabotka i aprobatsiia oprosnika otnosheniia k tekhnologiiam dlia podrost-kov i roditelei [Psychodiagnostics of technophobia and technophilia: Development and testing a questionnaire of attitudes towards technology for adolescents and parents]. Sotsial’naia psikhologiia i obshchestvo [Social Psychology and Society], 12(4), 170–188. 10.17759/sps.2021120410

[ref46] Soldatova, G.U., & Rasskazova, E.I. (2023). Tsifrovaia sotsializatsiia rossiiskikh podrostkov: skvoz’ prizmu sravneniia s podrostkami 18 evropeiskikh stran [Digital socialization of Russian teenagers: Th rough the prism of comparison with teenagers from 18 European countries]. Sotsial’naia psikhologiia i obshchestvo [Social Psychology and Society], 14(3), 11–30. 10.17759/sps.2023140302

[ref47] Soldatova, G.U., Chigarkova, S.V., Dreneva, A.A., & Koshevaya, A.G. (2020a). Effekt Iuliia Tsezaria: tipy mediamnogozadachnosti u detei i podrostkov [The Julius Caesar Effect: Types of media multitasking in children and adolescents]. Voprosy Psychologii [Issues of Psychology], 66(4), 54.

[ref48] Soldatova, G.U., Chigarkova, S.V., Koshevaya, A.G., & Nikonova, E.Yu. (2022). Povsednevnaia deiatel’nost’ podrostkov v smeshannoi real’nosti: pol’zovatel’skaia aktivnost’ i mnogozadachnost’ [Daily activities of adolescents in mixed reality: User activity and multitasking]. Sibirskii psikhologicheskii zhurnal [Siberian Psychological Journal], 83, 20–45. 10.17223/17267080/83/2

[ref49] Soldatova, G.U., Nikonova, E.Y., Koshevaya, A.G., & Trifonova, A.V. (2020b). Mediamnogozadachnost’: ot kognitivnykh funktsii k tsifrovoi povsednevnosti [Media multitasking: From cognitive functions to digital everyday life]. Sovremennaia zarubezhnaia psikhologiia [Journal of Modern Foreign Psychology], 9(4), 8–21. 10.17759/jmfp.2020090401

[ref50] Terry, C.A., Mishra, P., & Roseth, C.J. (2016). Preference for multitasking, technological dependency, student metacognition, & pervasive technology use: An experimental intervention. Computers in Human Behavior, 65, 241–251. 10.1016/j.chb.2016.08.009

[ref51] Tretyakova, V.S., & Tserkovnikova, N.G. (2021). Tsifrovoe pokolenie: poteri i priobreteniia [The digital generation: Losses and gains]. Professional’noe obrazovanie i rynok truda [Vocational Education and the Labour Market], 2, 53–65. 10.52944/PORT.2021.45.2.004

[ref52] Uvarov, A.Yu. (2018). Tekhnologii virtual’noj real’nosti v obrazovanii [Virtual reality technologies in education]. Nauka i shkola [Science and School], 4, 108–117.

[ref53] van der Schuur, W.A. , Baumgartner, S.E. , & Sumter, S.R. (2019). Social media use, social media stress and sleep: Examining cross-sectional and longitudinal relationships in adolescents. Health Communication, 34(5), 552–559. 10.1080/10410236.2017.1422101 29313723

[ref54] van der Schuur, W.A. , Baumgartner, S.E. , Sumter, S.R. , & Valkenburg, P.M. (2018). Media multitasking and sleep problems: A longitudinal study among adolescents. Computers in Human Behavior, 81, 316–324. 10.1016/j.chb.2017.12.024

[ref55] Vygotsky, L.S. (1960). Razvitie vysshikh psikhicheskikh funktsii [Development of higher mental functions].

[ref56] Wammes, J.D., Ralph, B.C., Mills, C., Bosch, N., Duncan, T.L., & Smilek, D. (2019). Disengagement during lectures: Media multitasking and mind wandering in university classrooms. Computers & Education, 132, 76–89. 10.1016/j.compedu.2018.12.007

[ref57] Wu, J.Y., & Xie, C. (2018). Using time pressure and note-taking to prevent digital distraction behavior and enhance online search performance: Perspectives from the load theory of attention and cognitive control. Computers in Human Behavior, 88, 244–254. 10.1016/j.chb.2018.07.008

[ref58] Zeer E.F., Tretyakova V.S., & Miroshnichenko V.I. (2019). Strategicheskie orientiry podgotovki pedagogicheskikh kadrov dlia sistemy nepreryvnogo professional’nogo obrazovaniia [Strategic directions of pedagogical personnel training for the system of continuing vocational education]. Obrazovanie i nauka [The Education and Science Journal], 6(21): 93–121. 10.17853/1994-5639-2019-6-93-121

